# LncRNATUG1 Facilitates Th2 Cell Differentiation by Targeting the miR-29c/B7-H3 Axis on Macrophages

**DOI:** 10.3389/fimmu.2021.631450

**Published:** 2021-07-16

**Authors:** Huiming Sun, Ting Wang, Weili Zhang, Heting Dong, Wenjing Gu, Li Huang, Yongdong Yan, Canhong Zhu, Zhengrong Chen

**Affiliations:** Department of Respiratory Medicine, Children’s Hospital of Soochow University, Suzhou, China

**Keywords:** bronchial asthma, long non-coding RNA taurine upregulated 1, miRNA-29c, B7-H3, T helper cell, macrophages

## Abstract

The role of long non-coding RNAs (lncRNA) in asthma remains unclear. In this study, we examined the role of long non-coding RNA taurine upregulated 1 (lncRNA TUG1) in asthma. We found that lncRNA TUG1 is one of the differentially expressed lncRNAs in the monocytes of asthmatic children and is associated with Th cell differentiation. LncRNA TUG1 and miR-29c are mainly distributed in the cytoplasm of macrophages. Our data suggested that lncRNA TUG1 increased in macrophages stimulated by House Dust Mite in a dose-dependent manner. Using loss- and gain of function strategy, we found that miR-29c might regulate Th2 cell differentiation by directly targeting co-stimulatory molecule B7-H3. Furthermore, down-regulation of lncRNA TUG1 decreased the level of GATA3 in CD4+T cells and was associated with miR-29c/B7-H3 axis. Moreover, the dual-luciferase reporter assay confirmed that lncRNA TUG1 serves as a competing endogenous RNA to sponge miR-29c. According to the rescue experiment, lncRNA TUG1 regulated Th2 cell differentiation *via* miR-29c. These data suggest that lncRNA TUG1 in macrophages regulates Th2 cell differentiation *via* miR-29c/B7-H3 axis.

## Introduction

Asthma is the most prevalent chronic respiratory disease worldwide. More than 300 million cases have been registered to date (1). Asthma is also the most common chronic respiratory disease in children. Over recent years, the prevalence of asthma symptoms has significantly increased in children and adolescents, particularly in developing countries, causing a consistent burden on the public health system (2).

Asthma is characterized by bronchial hyper-reactivity, mucus overproduction, airway inflammation, airway remodeling, and airway narrowing (3). T helper (Th) cells play a vital role in asthma. Th2 major cytokine (IL-4) production has been associated with allergic asthma and Th17 cytokine (IL-17) to neutrophilic asthma (4). Although many studies have focused on Th2 immune responses, there are still significant gaps in regulating Th2/Th17 cell differentiation and cytokine secretion. In our previous study, we found that costimulatory molecule B7-H3, which is regulated by miRNA-29c (miR-29c), is highly expressed on macrophages and plays an important role in Th2 cell differentiation (5). However, the mechanism of regulation of miR-29c in macrophages is still not fully understood.

The increasing evidence supports that long non-coding RNA (lncRNAs) has an important role in Th cell differentiation (6). Zhu et al. found that LncGAS5 derived from allergic rhinitis epithelium is the key mediator in Th1/Th2 differentiation (7). In addition, Chen and colleagues found that lnc-M2 could control the process of M2 macrophage differentiation *via* the PKA/CREB pathway (8). A recent study demonstrated that lncRNA TUG1 promotes proliferation and invasion and suppresses apoptosis in melanoma cells by sponging miR-29c-3p (9). However, the role of lncRNA TUG1 in macrophage-Th cell communication remains unclear.

In the present study, we investigated the effect of lncRNA on the miR-29c/B7-H3 axis in macrophages and the role of Th cell differentiation. We also detected the expression of lncRNA, miR-29c, and B7-H3 in children with asthma exacerbation. Our data demonstrated that lncRNA-TUG1 could regulate B7-H3 expression in macrophages by sponging miR-29c, which then controls Th2 differentiation.

## Materials and Methods

### Patients

Patients diagnosed with asthma according to the guidelines of the Global Initiative for Asthma (10) were enrolled from the department of pulmonary medicine at the Children’s Hospital of Soochow University. In addition, non-atopic healthy subjects were included as a control group. Peripheral blood samples from 3 patients with asthma exacerbation and 3 healthy children were used for lncRNA sequencing. In addition, 10 children with asthma exacerbation were recruited, and peripheral blood samples of acute or convalescent phase were collected for subsequent qPCR detection. All the subjects were instructed to avoid the use of asthma medication (such as glucocorticoids) prior to testing.

The present study was approved by the local Ethics Committee of the Children’s Hospital of Soochow University. All participants provided written informed consent.

### Isolation of Monocytes From Peripheral Blood

Peripheral blood mononuclear cells (PBMCs) were isolated from 2 ml of heparin-anticoagulated venous blood using Ficoll Histopaque (Sigma-Aldrich, St Louis, MO, USA) according to the manufacturer’s protocol. The monocytes were isolated from PBMCs using a negative selection kit (Miltenyi Biotec, Bergisch Gladbach, Germany). The procedure was described in a previous study (11).

### LncRNA Sequencing and Analysis

PBMC samples from asthmatic children and healthy controls were used for RNA sequencing to obtain lncRNA expression profiles. After total RNA was isolated, rRNAs were removed usingRibo-Zero rRNA Removal Kits (Illumina, San Diego, CA, USA) following the manufacturer’s instructions. RNA libraries were constructed by using rRNA-depleted RNAs with Tru-Seq Stranded Total RNA Library Prep Kit (Illumina, San Diego, CA, USA) according to the manufacturer’s instructions. Libraries were controlled for quality and quantified using the BioAnalyzer 2100 system (Agilent Technologies, Inc., USA). A total of 10pM libraries were denatured as single-stranded DNA molecules, captured on Illumina flow cells, amplified *in situ* as clusters, and finally sequenced for 150 cycles on Illumina Hi-Seq Sequencer according to the manufacturer’s instructions. Paired-end reads were harvested from Illumina HiSeq Sequencer after quality filtering. After 3’ adaptor-trimming by cutadapt software (v1.9), the high-quality trimmed reads were aligned to the reference genome (UCSC hg19) guided by the Ensembl GFF gene annotation file with TopHat2 software. Then, cuffdiff software (part of cufflinks) was used to get the gene-level FPKM as the expression profiles of lncRNAs; fold change and q-value were calculated based on FPKM. Finally, differentially expressed lncRNAs (fold change ≥2, P < 0.05) were identified. GO enrichment analyses were performed based on the differentially expressed lncRNAs.

### Culture of House Dust Mite (HDM)-Stimulated Macrophages

THP-1 cells (ATCC, Manassas, VA, USA) were seeded at 1×10^5^ cells/dish. Cells were incubated with phorbol myristate acetate (PMA, 50 ng/ml) for 6h. After that, THP-1 was cultured with medium without PMA for another 72h to induce THP-1 differentiation into macrophages. HDM extract (Dermatophagoides pteronyssinus; Stallergenes Greer, Lenoir, USA) with different concentrations (0.2 µg/ml or 2 µg/ml) was used to stimulate the macrophages for another 48h. Consequently, cells were collected to detect B7-H3 mRNA and protein levels by quantitative polymerase chain reaction (qPCR) and Western blot, respectively.

### Lentivirus Constructs

The over-expression and knockdown lentiviral vectors for miR-29c and B7-H3 were constructed by NOVOBIO Company (Shanghai, China). Empty vectors were used as a control. Recombinant plasmids were named LV210_pl-miR-29c (pLenti-miR-29c), LV289_pL6.3-GFP-TuDS-hsa-miR-29c (anti-miR-29c), VL1112-PDS159-B7H3 (pLenti-B7-H3), and VL1113-6_PDS019-B7H3-SH6 (anti-B7-H3). Lentiviral particles were produced in HEK293T cells as previously described (12). Briefly, lentiviral vectors encoding miR-29c or B7-H3 and packaging mix were co-infected into HEK293T cells. Then, the supernatant of HEK293Tcells were collected 48 hours after lentiviral vector transfection, and the concentration of the harvested virus was calculated by fluorescence ratio using flow cytometry.

### Macrophages Transfection and Th Cells Co-culture

100 nM pLenti-miR-29c or anti-miR-29c with or without pLenti-B7-H3 or anti-B7-H3 was transfected into THP-1 (2×10^5^/ml) for 48h. Then THP-1 cells (1×10^5^/ml) were seeded in a six-well plate, and cultured with RPMI 1640 medium (Life Technologies, Carlsbad, CA, USA) primed with 50 ng/ml PMA (Sigma, Louis, MO, USA) for 48h to induce macrophage differentiation. Meanwhile, naive CD4+ T cells (1×10^6^) were isolated from peripheral blood of healthy persons (donate fromRed Cross, Suzhou, China), and then collected and cultured for another 24h. Then, the CD4^+^ T cells were collected for further analysis.

### RNA-Fluorescence *In Situ* Hybridization (FISH)

Cells were incubated with 0.2 mol/L HCl for 0.5 h after being fixed with 4% formaldehyde for 20 min and then incubated with 5 μg/mL proteinase K for 15 min. Acetylation was performed with a specific solution including miR-29c-3p probe2μl, lncRNA-TUG1 probe 5μl, Buffer E 70μl, and DEPC 23μl, hybridized with FITC labeled lncRNA TUG1 probe (5μg/mL) for 24 h. Subsequently, cells were washed twice with 2 × SSC detergent containing 0.01% Tween-20 at 55°C.

### Dual-Luciferase Reporter Assay

The lncRNA TUG1 fragment containing miR-29c binding sites was synthesized to generate wild-type (lncRNA TUG1-WT) or mutant type (lncRNA TUG1-Mut). The lncRNA TUG1-WT and lncRNA TUG1-Mut fragments were subcloned into the firefly luciferase gene pGL3-Luciferase reporter vectors (Promega, USA) to generated pGL3-LncRNATUG1-WT and pGL3-LncRNA TUG1-MUT vectors, respectively. After that, the above vectors were co-transfected with miR-29c mimic or negative control into HEK293T cells for 24 h. Finally, firefly and renilla luciferase activities were sequentially measured using dual-luciferase assays (Promega) 24 h after the transfection and evaluated by the Bio Tek™ Microplate Reader.

### Small Interfering lncRNA TUG1 Transfection

Three siRNAs targeting TUG1 were conducted and are presented in **Table S1**. In brief, siRNA oligo (GenePharma, Suzhou, China) were added into six-well plates with PMA-induced macrophages (2×105/well) and then treated by using GP-Transfect-Mate (GenePharma, Suzhou, China) according to the operation manual. After 24 or 48 h, macrophages were collected for further experiments. Then, qPCR analysis was applied to detect lncRNA TUG1 expression and to validate the transfected efficiencies. According to the interfering efficiencies, lncRNA TUG1 shRNA-1 was selected for the next experiment. In brief, lncRNA TUG1 shRNA-1 with or without miR-29c inhibitor (GenePharm, Suzhou, China) were added into macrophages for 6h and co-cultured with naive CD4^+^ T cells described above for another 24 h. After that, the supernatant in the lower chamber and CD4^+^ T cells in the upper chamber was collected.

### RNA Extraction and Quantitative Polymerase Chain Reaction (qPCR)

Total RNA was extracted from experiment cells such as PBMCs, macrophages, and CD4^+^ T cells using Trizol Reagent (Invitrogen, Carlsbad, USA) according to the manufacture’s protocol. RNA was revere-transcribed into cDNA using PrimeScript TM RT reagent kit (Takara, Japan). Revere transcript qPCR Master Mix was purchased from (Takara, Japan). The primer sequences are shown in **Table S2**.

### ELISA

The supernatants from cell cultures were collected, and all samples were preserved at −80°C for subsequent assay of soluble B7-H3 (sB7-H3) by ELISA as previously described (13). Meanwhile, IFN-γ, IL-4, and IL-17 were also detected by ELISA (R&D Systems, USA).

### Statistical Analysis

All data were expressed using mean ± standard deviation (± SD). SPSS 21.0 software (SPSS Inc., IL, USA) was used to conduct statistical analysis. Student’s t-test was used to analyze the differential expression of lncRNA TUG1, miR-29c, and B7-H3 mRNA between asthmatic children and controls. The comparisons among multiple groups were performed with a one-way analysis of variance followed by Dunnett’s test. P-value of <0.05 was considered to have statistical significance.

## Results

### Expression and Function Analysis of lncRNA TUG1 in Monocytes

**Figure 1:**
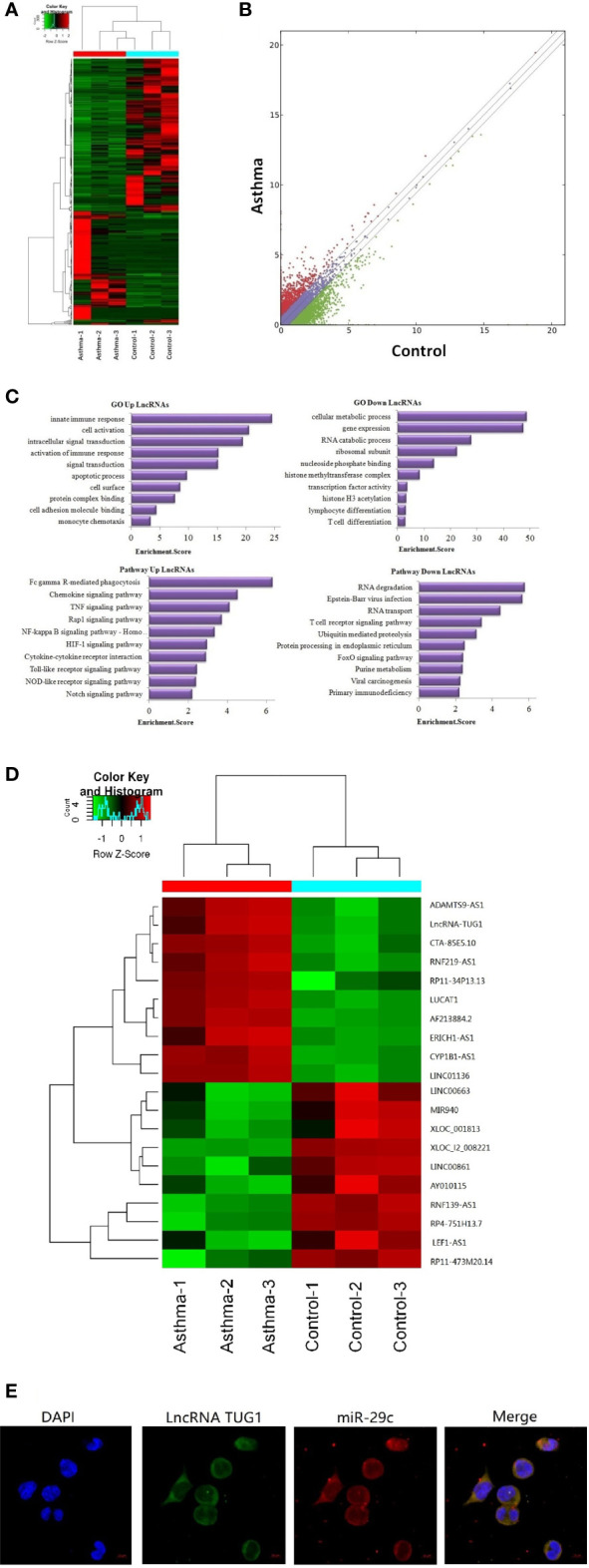
Sequencing of lncRNAs in monocytes and co-location with miR-29c in macrophages. **(A)** The heat map depicts the hierarchical clustering of altered lncRNAs in monocytes between children with asthma exacerbation (n = 3) and the healthy control group (n = 3). **(B)** The scatter plot reveals that a significant difference existed in the distribution of lncRNAs between asthma exacerbation and the healthy control group. **(C)** GO and KEGG signaling pathways for lncRNAs that are differentially downregulated and target mRNAs. **(D)** The heat map depicts the hierarchical clustering of the top 10 up-regulated and down-regulated lncRNAs. **(E)** LncRNA TUG1 and miR-29c mainly distributed in the cytoplasm of macrophages using the FISH analysis from three independent experiments.

A total of 23,709 lncRNAs were detected in the PBMCs collected from asthma and control patients. Compared to the control group, 163 lncRNAs were significantly up-regulated, and 226 lncRNAs were significantly down-regulated in patients with asthma (**Figures 1A, B**). Meanwhile, the mRNAs were also detected using RNA-seq (see the supplementary data).

Based on Gene Ontology (GO) analysis, the target mRNAs of this differentiated expressed lncRNA are involved in the T cell activation and T cell differentiation. According to the KEGG pathway analysis, the T cell receptor signaling pathway is one of the major pathways affecting T cell differentiation (**Figure 1C**).

The top 10 up-regulated and down-regulated lncRNAs are shown in **Figure 1D**. LncRNATUG1 was more than 5 fold higher in patients with asthma compared to the controls. The previous studies have demonstrated that lncRNATUG1 has an important role in promoting the development of pancreatic cancer and bladder cancer by sponging miR-29c (14, 15). However, no study has reported on the function of lncRNA TUG1 in macrophages and T cell differentiation. We examined the cellular location of lncRNA TUG1 and miR-29c in monocytes by RNA FISH analysis and found that both of them were mainly distributed in the cytoplasm of macrophages (**Figure 1E**), which suggested a potential role of the lncRNA TUG1/miR-29c axis in regulating the activation of the human macrophages.

### LncRNA TUG1 Was Induced in the HDM-Stimulated Macrophages

**Figure 2:**
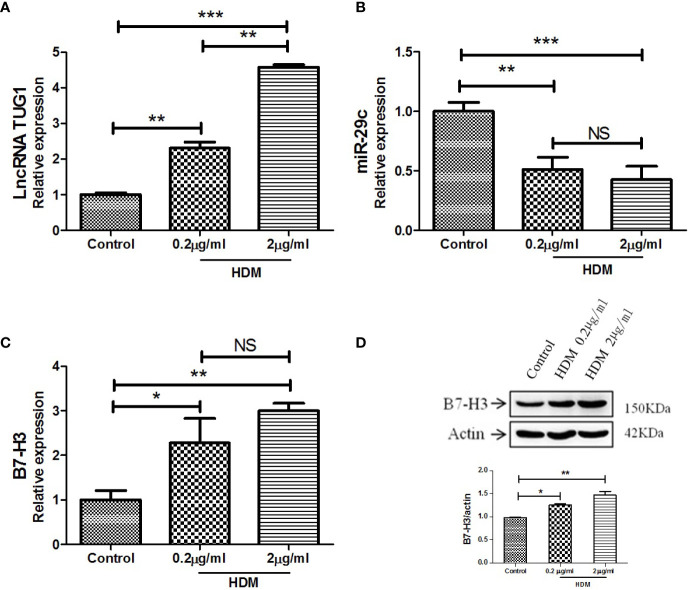
LncRNA TUG1 induced by HDM in macrophages in a dose-dependent manner. **(A)** LncRNA TUG1 was increased in macrophages stimulated by HDM in a dose-dependent manner. **(B)** The level of miR-29c significantly decreased, but it was not dose-dependent. **(C, D)** HDM also could both induce B7-H3 expression at mRNA and protein levels in a dose-dependent manner. Data are shown as Mean ± SD and represent three independent experiments. *p < 0.05, **p < 0.01 and ***p < 0.001. NS, no significance.

As shown in **Figure 2A**, LncRNA TUG1 was increased in the macrophages stimulated by HDM in a dose-dependent manner. Meanwhile, the level of miR-29c significantly decreased even at the low dose of HDM stimulation (**Figure 2B**). As for B7-H3, HDM could induce B7-H3 expression at mRNA and protein levels in a dose-dependent manner (P < 0.05) (**Figures 2C, D**). Taken together, our results suggest a negative correlation between lncRNA TUG1 and miR-29c, a previously described suppressor of B7-H3, during asthma progression.

### Macrophages Regulate Th2 Cell Differentiation *via* miR-29c/B7-H3 Pathway

**Figure 3:**
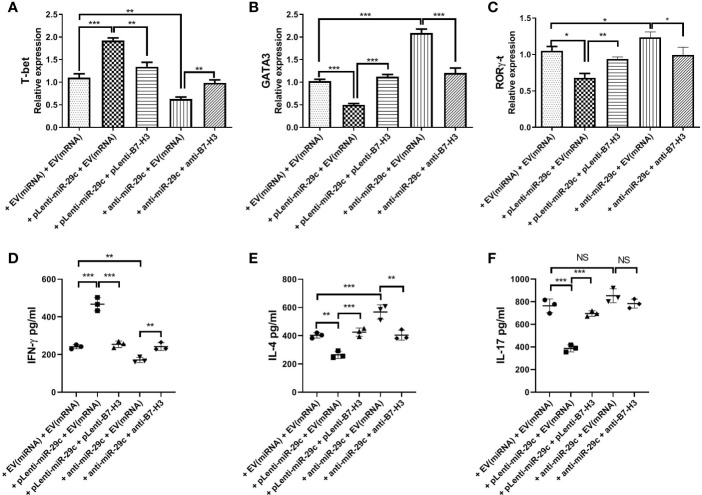
Macrophages regulated Th2 cell differentiation *via* miR-29c/B7-H3 pathway. Using loss and gain of function strategy, 100 nMpLenti-miR-29c or anti-miR-29c was transfected into macrophages for 48h, and then pLenti-B7-H3 or anti-B7-H3 was added into the culture in the lower chambers of transwell for 24h. After that, RNA was extracted, and transcription factors were detected by qPCR and IFN-γ, IL-4 and IL-17 were detected by ELISA. **(A)** Relative expression of T-bet. **(B)** Relative expression of GATA3. **(C)** Relative expression of RORγ-t. **(D)** Levels of IFN-γ. **(E)** Levels of IL-4.** (F)** Levels of IL-17. Data shown as Mean ± SD and represent three independent experiments. *p < 0.05, **p < 0.01 and ***p < 0.001. NS, no significance.

To confirm the possibility that miR-29c-mediated suppression of B7-H3 on macrophages leads to Th cell polarization, macrophages were co-cultured with naive CD4^+^ T cells since the membrane B7-H3 and soluble B7-H3 have a different function on T cell activation and proliferation (16). Using loss- and gain-of-function strategy, pLenti-miR-29c or anti-miR-29c was transfected into macrophages. Then, pLenti-B7-H3 or anti-B7-H3 was added into the culture to evaluate the function of the miR-29c/B7-H3 axis in regulating Th cell differentiation. As shown in **Figure 3**, over-expression of miR-29c could decrease the level of GATA3 and RORγ-t and increase the T-bet in CD4^+^T cells (P < 0.05). Meanwhile, down-regulation of miR-29c could increase GATA3 and RORγ-t and decrease the T-bet in CD4^+^T cells (P < 0.05). The expression of GATA3, T-bet, and RORγ-t could be rescued by pLenti-B7-H3 or anti-B7-H3 (P < 0.05)(**Figure 3**). Meanwhile, the cytokines of IFN-γ, IL-4, and IL-17 were detected by ELISA. We found that over-expression of miR-29c could decrease IL-4 and IL-17 while down-regulation of miR-29c has the opposite effect (**Figure 3**). Taken together, these results suggested that miR-29c regulates Th2 cell differentiation by targeting co-stimulatory molecule B7-H3 in macrophages.

### Knockdown of lncRNATUG1 Decreases the Level of GATA3 in CD4^+^T Cells

**Figure 4:**
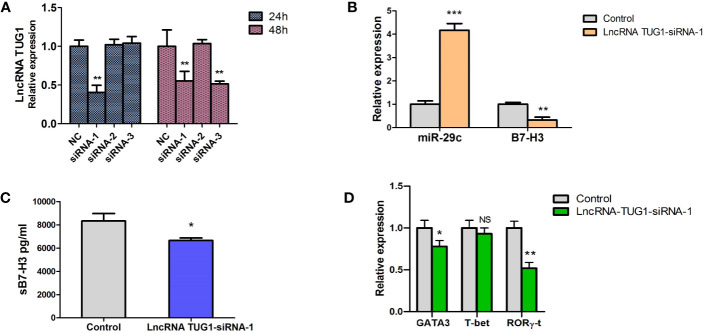
Down-regulation of LncRNA TUG1 decreased the level of GATA3 in CD4+T cells and was associated with miR-29c/B7-H3 axis. **(A)** We constructed 3 siRNAs of LncRNA TUG1. Three siRNAs were added into macrophages and detected LncRNA TUG1 at 24h and 48h. **(B)** TUG1-siRNA-1 was added into co-culture of macrophages and naive CD4+ T cells, and miR-29c and B7-H3 in macrophages were detected by qPCR. **(C)** The supernatant was collected to detect sB7-H3 using ELISA. **(D)** CD4+T cells after co-culturing were collected, and the levels of GATA3 and RORγ-t in CD4+ T cells were detected by qPCR. Data were shown as Mean ± SD and represent three independent experiments. *p < 0.05, **p < 0.01 and ***p < 0.001. NS, no significance.

To study the function of lncRNA TUG1 in the regulation of Th cell differentiation, we constructed 3 siRNAs of lncRNA TUG1. Compared to other siRNAs, siRNA-1 showed a better interfering effect at 24h and 48h (P < 0.05) (**Figure 4A**). To determine whether lncRNA TUG1 regulates Th cell differentiation through the miR-29c/B7-H3 axis, lncRNA TUG1-siRNA-1 was transfected into the macrophages and co-cultured with naive CD4^+^ T cells. As shown in **Figure 4**, lncRNATUG1-siRNA-1 increased the expression of miR-29c and decreased the expression of B7-H3 mRNA and protein (P < 0.05) (**Figures 4B, C**). Simultaneously, the expression of GATA3 and RORγ-t in CD4^+^ T cells decreased (P < 0.05) after lncRNATUG1-siRNA-1 transfection (**Figure 4D**). These results show that lncRNA TUG1 positively regulates Th cell differentiation by potentially targeting miR-29c/B7-H3 axis.

### LncRNA TUG1 Serves as a ceRNA to Sponge miR-29c

**Figure 5:**
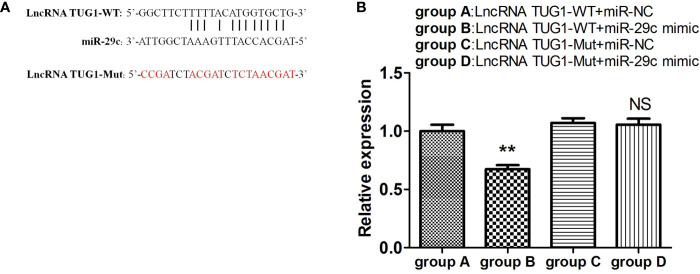
LncRNA TUG1 serves as a ceRNA to sponge miR-29c. **(A)** The putative binding sites between LncRNA TUG1-WT and miR-29c. The sequence of LncRNA TUG1-Mut was also shown. **(B)** The LncRNA TUG1-WT and LncRNA TUG1-Mut fragments were subcloned into the Renilla luciferase gene pGL3-Luciferase reporter vectors (Promega, USA) to generated pGL3-LncRNATUG1-WT and pGL3-LncRNA TUG1-MUT vectors, respectively. After that, the above vectors were co-transfected with miR-29c mimic or negative control into 293T cells for 24 h, and then the luciferase activities were detected. Data are shown as Mean ± SD and represent three independent experiments. **p < 0.01. NS, no significance.

To investigate the interaction between lncRNA TUG1 and miR-29c, the starBase online website was employed to search the binding sites, and the binding sites are shown in **Figure 5A**. To further validate the interaction of lncRNA TUG1 and miR-29c, lncRNA TUG1-WT reporter containing predicted binding sites of miR-29c and lncRNA TUG1-Mut reporter with mutant binding sites of miR-29c were constructed. As shown in **Figure 5**, the luciferase activities were reduced after co-transfection of lncRNA TUG1-WT and miR-29c mimics into HEK293T cells (P < 0.01). However, no difference in luciferase activity was found between lncRNA TUG1-Mut groups (P > 0.05)(**Figure 5B**), suggesting that lncRNA TUG1 interacts with miR-29c using putative binding sites. A recent study confirmed that lncRNA TUG1 could sponge miR-29c directly in Sk-Hep-1 and HeP3B cells using an RNA-binding protein immunoprecipitation assay (17). Taken together, lncRNA TUG1 serves as a ceRNA to sponge endogenous miR-29c.

### LncRNA TUG1 Facilitates Th2 Cell Differentiation by Inhibiting miR-29c

**Figure 6:**
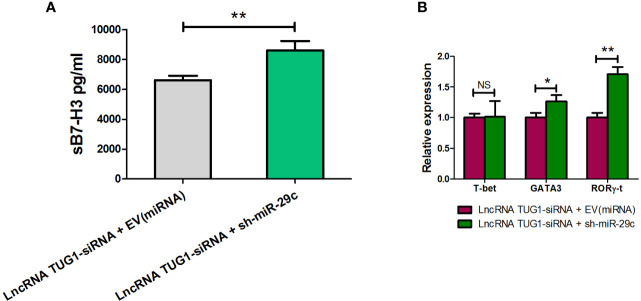
LncRNA TUG1 regulated Th cell differentiation *via* miR-29c. **(A)** TUG1 siRNA-1 with or without miR-29c inhibitor (GenePharm, Suzhou, China) were transfected into macrophages for 6h and co-cultured with naive CD4+ T cells described above for another 24 h. After that, the supernatant in the lower chamber and CD4+ T cells in the upper chamber was collected for sB7-H3 detection. **(B)** CD4+T cells after co-culture were collected, and the levels of T-bet, GATA3, and RORγ-t in CD4+ T cells were detected by qPCR. Data are shown as mean ± SD and represent three independent experiments. *p < 0.05 and **p < 0.01. NS, no significance.

To confirm the mechanism of Th cell differentiation regulated by lncRNA TUG1, we performed rescue experiments. As shown in **Figure 6**, down-regulation of miR-29c could reverse the suppressive effect of sB7-H3 by lncRNA TUG1-siRNA-1 in macrophages and CD4^+^ T cells co-culture (P < 0.05). Meanwhile, down-regulation of miR-29c in macrophages increased the expression of GATA3 and RORγ-t in CD4^+^ T cells (P < 0.05) using the transwell co-culture system described above. These data suggest that lncRNA TUG1 may facilitate Th2 cell differentiation by inhibiting miR-29c.

### Expression of lncRNA TUG1, miR-29c, and B7-H3 in Peripheral Blood in Children With Asthma Exacerbation

**Figure 7:**
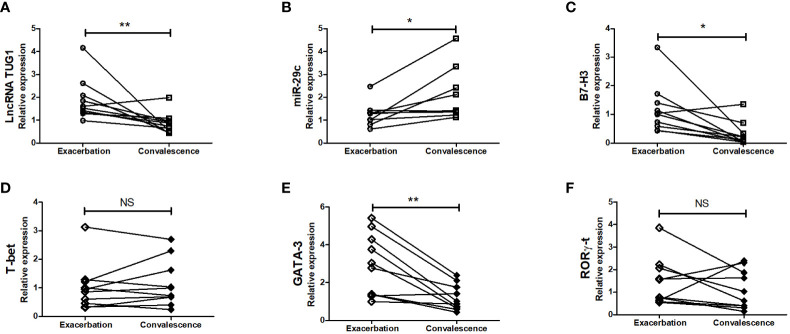
Verification of the expression of LncRNA TUG1, miR-29c, and B7-H3 in asthmatic children. We determined the relative expression of LncRNA TUG1 (n = 10). **(A)**, miR-29c **(B)** and B7-H3 **(C)** T-bet **(D)**, GATA-3 **(E)**, RORγ-t **(F)** in peripheral blood in 10 children with asthma exacerbation and in convalescent-phase by qPCRs. *p < 0.05 and **p < 0.01. NS, no significance.

To further examine whether lncRNA TUG1, miR-29c, and B7-H3 participate in asthma exacerbation, we measured the expression of lncRNA TUG1, miR-29c, and B7-H3 in the peripheral blood collected from 10 children with asthma exacerbation and in the convalescent phase. As shown in **Figure 7**, lncRNA TUG1 and B7-H3 decreased in the convalescent phase compared to acute exacerbation, while miR-29c increased in the convalescent-phase (P < 0.05), which presumed that lncRNA TUG1/miR-29c/B7-H3 axis participates in the asthma exacerbation.

## Discussion

LncRNA is a type of non-coding RNA with 200 nucleotides (18). Recent studies have indicated that lncRNAs have the function of sponging miRNA and play an important role in lung diseases such as chronic obstructive pulmonary disease, cancer, and asthma (19). LncRNAs regulate epigenetic, transcriptional, and translational responses, and are critical regulators of immune cell differentiation, especially for type-2 immune responses (20, 21).

Previous studies have suggested that lncRNA TUG1 participates in the development of cancers (22, 23), hypoxic pulmonary hypertension (24), and Alzheimer’s disease (25). Moreover, the experimental asthma model suggested that lncRNA TUG1 promotes airway smooth muscle cell proliferation and migration by sponging miR-590-5p/FGF1 (26). In this study, we further examined the role of lncRNA TUG1 in asthma pathogenesis, including Th2 and Th17 cell differentiation. We found that lncRNA TUG1 and B7-H3 were up-regulated in children with asthma exacerbation, and miR-29c was down-regulated compared to controls. Moreover, lncRNA TUG1 was increased in macrophages stimulated by HDM *in vitro* in a dose-dependent manner. Furthermore, our data indicated that lncRNA TUG1 interacted with miR-29c by putative binding sites in the cytoplasm. Moreover, we found that miR-29c might regulate Th2 cell differentiation by directly targeting co-stimulatory molecule B7-H3. Meanwhile, the down-regulation of lncRNA TUG1 decreased the level of GATA3 in CD4+T cells and was associated with miR-29c/B7-H3 axis.

LncRNA TUG1 has other miRNA targets, including miR-92a (27), miR-143-5p (28), miR-590-5p (29), et al., which suggests that lncRNAs could mediate sponge regulatory network of gene expression, which is confirmed in prostate cancer (30).

In summary, the present study found the novel mechanism of LncRNA TUG1 in the regulation of Th2 cell differentiation *via* the miR-29c/B7-H3 axis in macrophages. This study provides novel potential diagnostic biomarkers and therapeutic targets for asthma. However, further studies should be conducted to verify the effect of LncRNA TUG1 and miR-29c as therapeutic targets in terms of the experimental asthma model *in vivo*.

## Data Availability Statement

The original contributions presented in the study are included in the article/**Supplementary Material**. Further inquiries can be directed to the corresponding authors.

## Ethics Statement

The present study was approved by the local Ethics Committee of Children’s Hospital of Soochow University. Written informed consent to participate in this study was provided by the participants’ legal guardian/next of kin. Written informed consent was obtained from the individual(s), and minor(s)’ legal guardian/next of kin, for the publication of any potentially identifiable images or data included in this article.

## Author Contributions

HS, TW and WZ performed experiments, analyzed data, and wrote the manuscript. ZC and CZ designed the study, analyzed data, and revised the manuscript. HD and WG analyzed data. LH and YY collected the clinical data. All authors read and approved the final manuscript. All authors contributed to the article and approved the submitted version.

## Funding

This study was supported by the National Science Foundation of China (grant to ZC, no.81771676 and 81970027; grant to LH, no. 81971490; grant to Yongdong Yan, no. 81870006), the Social Development Projects of Jiangsu Province (grant to ZC, no. BE2019671), Jiangsu Provincial Medical Youth Talent (grant NO. QNRC2016766), Suzhou Medical Youth Talent (grant NO. GSWS2019047), and Suzhou Medical Technology Projects of Clinical Key Diseases (grant NO. LCZX201809).

## Conflict of Interest

The authors declare that the research was conducted in the absence of any commercial or financial relationships that could be construed as a potential conflict of interest.

## Supplementary Material

Supplementary Table 1 The sequences of TUG1-siRNAs.

Supplementary Table 2 The primers of PCRs.

## References

[1] Collaborators GBDCRD. Global, Regional, and National Deaths, Prevalence, Disability-Adjusted Life Years, and Years Lived With Disability for Chronic Obstructive Pulmonary Disease and Asthma, 1990-2015: A Systematic Analysis for the Global Burden of Disease Study 2015. Lancet Respir Med (2017) 5(9):691–706. 10.1016/S2213-2600(17)30293-X 28822787 PMC5573769

[2] Ferrante G La Grutta S. The Burden of Pediatric Asthma. Front Pediatr (2018) 6:186. 10.3389/fped.2018.00186 29988370 PMC6023992

[3] Lambrecht BN Hammad H. The Immunology of Asthma. Nat Immunol (2015) 16(1):45–56. 10.1038/ni.3049 25521684

[4] Boonpiyathad T Sozener ZC Satitsuksanoa P Akdis CA. Immunologic Mechanisms in Asthma. Semin Immunol (2019) 46:101333. 10.1016/j.smim.2019.101333 31703832

[5] Zhang X Zhao X Sun H Yan Y Huang L Gu W. The Role of miR-29c/B7-H3 Axis in Children With Allergic Asthma. J Trans Med (2018) 16(1):218. 10.1186/s12967-018-1590-8 PMC6076420 30075787

[6] West KA Lagos D. Long Non-Coding Rna Function in CD4(+) T Cells: What We Know and What Next? Non-coding RNA (2019) 5(3):43. 10.3390/ncrna5030043 PMC6789709 31336952

[7] Zhu X Wang X Wang Y Zhao Y. Exosomal Long non-Coding RNA GAS5 Suppresses Th1 Differentiation and Promotes Th2 Differentiation *Via* Downregulating EZH2 and T-bet in Allergic Rhinitis. Mol Immunol (2020) 118:30–9. 10.1016/j.molimm.2019.11.009 31841965

[8] Chen Y Li H Ding T Li J Zhang Y Wang J. Lnc-M2 Controls M2 Macrophage Differentiation *Via* the PKA/CREB Pathway. Mol Immunol (2020) 124:142–52. 10.1016/j.molimm.2020.06.006 32563859

[9] Wang Y Liu G Ren L Wang K Liu A. Long non-Coding RNA TUG1 Recruits miR29c3p From Its Target Gene RGS1 to Promote Proliferation and Metastasis of Melanoma Cells. Int J Oncol (2019) 54(4):1317–26. 10.3892/ijo.2019.4699 30720136

[10] Rajan S Gogtay NJ Konwar M Thatte UM. The Global Initiative for Asthma Guidelines (2019): Change in the Recommendation for the Management of Mild Asthma Based on the SYGMA-2 Trial - a Critical Appraisal. Lung India (2020) 37(2):169–73. 10.4103/lungindia.lungindia_308_19 PMC7065541 32108606

[11] Shi H Chen L Ridley A Zaarour N Brough I Caucci C. Gm-Csf Primes Proinflammatory Monocyte Responses in Ankylosing Spondylitis. Front Immunol (2020) 11:1520. 10.3389/fimmu.2020.01520 32765525 PMC7378736

[12] Su Z Wu F. Inflammatory Factors Induce Thrombosis Through the miR-146b-3p/p38MAPK/COX-2 Pathway. BioMed Res Int (2020) 2020:8718321. 10.1155/2020/8718321 32337281 PMC7154971

[13] Zhang G Hou J Shi J Yu G Lu B Zhang X. Soluble CD276 (B7-H3) Is Released From Monocytes, Dendritic Cells and Activated T Cells and Is Detectable in Normal Human Serum. Immunology (2008) 123(4):538–46. 10.1111/j.1365-2567.2007.02723.x PMC2433324 18194267

[14] Lu Y Tang L Zhang Z Li S Liang S Ji L. Long Noncoding Rna TUG1/miR-29c Axis Affects Cell Proliferation, Invasion, and Migration in Human Pancreatic Cancer. Dis Markers (2018) 2018:6857042. 10.1155/2018/6857042 30595764 PMC6282130

[15] Guo P Zhang G Meng J He Q Li Z Guan Y. Upregulation of Long Noncoding RNA Tug1 Promotes Bladder Cancer Cell Proliferation, Migration, and Invasion by Inhibiting Mir-29c. Oncol Res (2018) 26(7):1083–91. 10.3727/096504018X15152085755247 PMC7844683 29321088

[16] Sun J Fu F Gu W Yan R Zhang G Shen Z. Origination of New Immunological Functions in the Costimulatory Molecule B7-H3: The Role of Exon Duplication in Evolution of the Immune System. PLoS One (2011) 6(9):e24751. 10.1371/journal.pone.0024751 21931843 PMC3172298

[17] Zhao W Jiang X Yang S. Lncrna TUG1 Promotes Cell Proliferation, Migration, and Invasion in Hepatocellular Carcinoma *Via* Regulating miR-29c-3p/COL1A1 Axis. Cancer Manag Res (2020) 12:6837–47. 10.2147/CMAR.S256624 PMC7425090 32821161

[18] Rinn JL Chang HY. Genome Regulation by Long Noncoding Rnas. Annu Rev Biochem (2012) 81:145–66. 10.1146/annurev-biochem-051410-092902 PMC3858397 22663078

[19] Li Y Yin Z Fan J Zhang S Yang W. The Roles of Exosomal miRNAs and lncRNAs in Lung Diseases. Signal Transduct Target Ther (2019) 4(1):47. 10.1038/s41392-019-0080-7 31728212 PMC6851157

[20] Guidi R Wedeles CJ Wilson MS. ncRNAs in Type-2 Immunity. Non-coding RNA (2020) 6(1):10. 10.3390/ncrna6010010 PMC7151598 32155783

[21] Xia L Wang X Liu L Fu J Xiao W Liang Q. lnc-BAZ2B Promotes M2 Macrophage Activation and Inflammation in Children With Asthma Through Stabilizing BAZ2B Pre-Mrna. J Allergy Clin Immunol (2020) 147(3):921–32. 10.1016/j.jaci.2020.06.034 32712329

[22] Shen X Hu X Mao J Wu Y Liu H Shen J. The Long Noncoding RNA TUG1 Is Required for TGF-beta/TWIST1/EMT-mediated Metastasis in Colorectal Cancer Cells. Cell Death Dis (2020) 11(1):65. 10.1038/s41419-020-2254-1 31988275 PMC6985237

[23] El-Khazragy N Mohammed HF Yassin M Elghoneimy KK Bayoumy W Hewety A. Tissue-Based Long non-Coding RNAs “Pvt1, TUG1 and MEG3” Signature Predicts Cisplatin Resistance in Ovarian Cancer. Genomics (2020) 112(6):4640–6. 10.1016/j.ygeno.2020.08.005 32781203

[24] Yang L Liang H Shen L Guan Z Meng X. Lncrna Tug1 Involves in the Pulmonary Vascular Remodeling in Mice With Hypoxic Pulmonary Hypertension *Via* the microRNA-374c-mediated Foxc1. Life Sci (2019) 237:116769. 10.1016/j.lfs.2019.116769 31422096

[25] Li X Wang SW Li XL Yu FY Cong HM. Knockdown of Long non-Coding RNA TUG1 Depresses Apoptosis of Hippocampal Neurons in Alzheimer’s Disease by Elevating microRNA-15a and Repressing ROCK1 Expression. Inflammation Res (2020) 69(9):897–910. 10.1007/s00011-020-01364-8 32577774

[26] Lin J Feng X Zhang J Tong Z. Long Noncoding RNA TUG1 Promotes Airway Smooth Muscle Cells Proliferation and Migration *Via* Sponging miR-590-5p/FGF1 in Asthma. Am J Trans Res (2019) 11(5):3159–66. 10.21203/rs.3.rs-15500/v1 PMC6556671 31217885

[27] Yang L Li T. Lncrna TUG1 Regulates ApoM to Promote Atherosclerosis Progression Through miR-92a/FXR1 Axis. J Cell Mol Med (2020) 24(15):8836–48. 10.1111/jcmm.15521 PMC7412710 32597038

[28] Yu X Hu L Li S Shen J Wang D Xu R. Long Non-Coding RNA Taurine Upregulated Gene 1 Promotes Osteosarcoma Cell Metastasis by Mediating HIF-1alpha *Via* Mir-143-5p. Cell Death Dis (2019) 10(4):280. 10.1038/s41419-019-1509-1 30911001 PMC6433912

[29] Ai Y Chen M Liu J Ren L Yan X Feng Y. Lncrna TUG1 Promotes Endometrial Fibrosis and Inflammation by Sponging miR-590-5p to Regulate Fasl in Intrauterine Adhesions. Int Immunopharmacol (2020) 86:106703. 10.1016/j.intimp.2020.106703 32599321

[30] Du Z Sun T Hacisuleyman E Fei T Wang X Brown M. Integrative Analyses Reveal a Long Noncoding RNA-Mediated Sponge Regulatory Network in Prostate Cancer. Nat Commun (2016) 7:10982. 10.1038/ncomms10982 26975529 PMC4796315

