# Supplementary material for: LncRNATUG1 Facilitates Th2 Cell Differentiation by Targeting the miR-29c/B7-H3 Axis on Macrophages
Source: Front Immunol. 2021 Jul 16;12:631450. doi: 10.3389/fimmu.2021.631450 (PMC8322941; doi:10.3389/fimmu.2021.631450)
Supplement: Supplementary Table 1 — The sequences of TUG1-siRNAs. [file Table_1.docx]

**Table S1.** The sequences of TUG1-siRNAs

|  | Sense | Antisense |
| --- | --- | --- |
| TUG1-homo-3522  siRNA-1 | 5' GCUACAACUAUCUUCCUUUTT 3' | 5' AAAGGAAGAUAGUUGUAGCTT 3' |
| TUG1-homo-718  siRNA-2 | 5' GCGAGUCACUCUGUAAUUUTT 3' | 5' AAAUUACAGAGUGACUCGCTT 3' |
| TUG1-homo-6117  siRNA-3 | 5' GCCUCUAUUCCUGUAUGUATT 3' | 5' UACAUACAGGAAUAGAGGCTT 3' |
