# Supplementary material for: LncRNATUG1 Facilitates Th2 Cell Differentiation by Targeting the miR-29c/B7-H3 Axis on Macrophages
Source: Front Immunol. 2021 Jul 16;12:631450. doi: 10.3389/fimmu.2021.631450 (PMC8322941; doi:10.3389/fimmu.2021.631450)
Supplement: Supplementary Table 2 — The primers of PCRs. [file Table_2.docx]

**Table S2.** The primers of PCRs

| **Gene** |  | **Sequence** |
| --- | --- | --- |
| **U6** | F primer | CAGCACATATACTAAAATTGGAACG |
|  | R primer | ACGAATTTGCGTGTCATCC |
|  | Size: 76bp | |
| **LncRNA TUG1** | F primer | GCATACTCCTTGACCAGCAACTT |
|  | R primer | ACTGAAGGAGAGAAATGGACGCG |
|  | Size: 160 bp | |
| **miR-29c** | F primer | CTCCTCCTTTTAGCACCATTTG |
|  | R primer | TATGCTTGTTCTCGTCTCTGTGTC |
|  | Size: 70 bp | |
| **B7H3** | F primer | CTGGCTTTCGTGTGCTGGAGA |
|  | R primer | GCTGTCAGAGTGTTTCAGAGGCT |
|  | Size: 126 bp | |
| **GATA-3** | F primer | GCAATGCCTGTGGGCTCTACT |
|  | R primer | CTTGGGGAAGTCCTCCAGTGA |
|  | Size: 149 bp | |
| **T-bet** | F primer | GCTCACAAACAACAAGGGGG |
|  | R primer | TATGCGTGTTGGAAGCGTTG |
|  | Size: 143 bp | |
| **RORγ-t** | F primer | AGCGCTCCAACATCTTCTCCC |
|  | R primer | CCACCACGTACTGAATGGCCT |
|  | Size: 114bp | |
